# The Conserved Sonic Hedgehog Limb Enhancer Consists of Discrete Functional Elements that Regulate Precise Spatial Expression

**DOI:** 10.1016/j.celrep.2017.07.037

**Published:** 2017-08-08

**Authors:** Laura A. Lettice, Paul Devenney, Carlo De Angelis, Robert E. Hill

**Affiliations:** 1 MRC Human Genetics Unit, MRC Institute of Genetics and Molecular Medicine, University of Edinburgh, Edinburgh EH4 2XU, UK

**Keywords:** *Shh* expression, limb development, ZRS, enhancer, *HoxD* genes, Werner mesomelic syndrome, polydactyly, genome editing, phenotype

## Abstract

Expression of sonic hedgehog (*Shh*) in the limb bud is regulated by an enhancer called the zone of polarizing activity regulatory sequence (ZRS), which, in evolution, belongs to an ancient group of highly conserved *cis* regulators found in all classes of vertebrates. Here, we examined the endogenous ZRS in mice, using genome editing to establish the relationship between enhancer composition and embryonic phenotype. We show that enhancer activity is a consolidation of distinct activity domains. Spatial restriction of *Shh* expression is mediated by a discrete repressor module, whereas levels of gene expression are controlled by large overlapping domains containing varying numbers of HOXD binding sites. The number of HOXD binding sites regulates expression levels incrementally. Substantial portions of conserved sequence are dispensable, indicating the presence of sequence redundancy. We propose a collective model for enhancer activity in which function is an integration of discrete expression activities and redundant components that drive robust expression.

## Highlights

• The ancient vertebrate enhancer, the ZRS, shows sequence plasticity
• Discrete regulatory activities are assigned to specific sites in the enhancer
• The number of HOXD binding sites determines the level of *Shh* expression
• Robust expression is a collective of regulatory and redundant information

## Graphical Abstract

**Figure:**
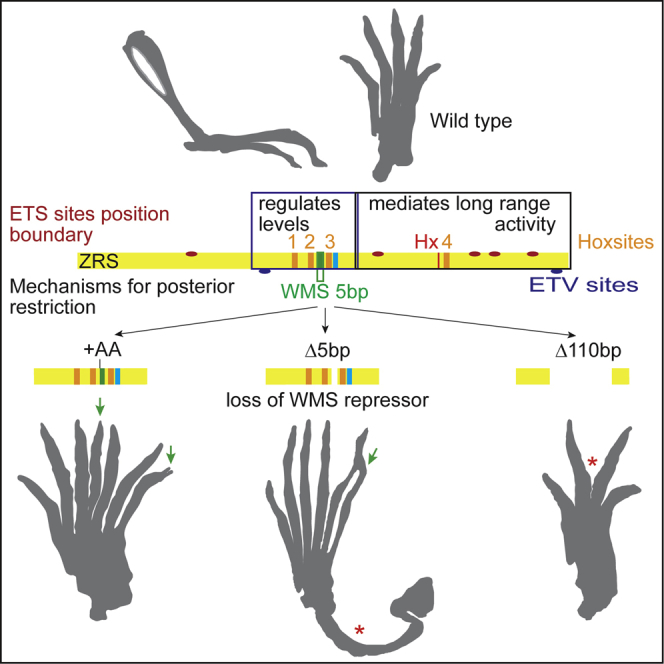

## Teaser

Lettice et al. examine the composition of a highly conserved limb-specific enhancer, the ZRS, by dissecting the endogenous sequence using genome editing. Analysis of the resulting phenotype gives insights into the complex composition of the enhancer, which integrates discrete expression activities and redundant elements to drive accurate spatiotemporal gene expression.

## Introduction

The basis of embryonic development lies in the spatiotemporal control of gene expression, which is mediated by remote *cis*-regulatory elements. These *cis*-acting elements, or enhancers, are fundamental to evolution and disease. Despite these important roles, major unanswered questions remain about the information encoded by the enhancer sequence and the importance of the overall structural architecture to enhancer activity. One class of enhancers that operates during embryogenesis are those that are highly conserved, acting at long distances from their target genes (Visel et al., 2009). Here we focus on a highly conserved element called the zone of polarizing activity regulatory sequence (ZRS) that is responsible for the spatiotemporal expression of *Shh* during limb bud development (Lettice et al., 2003, Sagai et al., 2005) and is essential for specifying digit identity and number. This enhancer is ∼770 bp in length and shows a high degree of similarity in vertebrates across a lengthy evolutionary timescale, including sharks and rays (Dahn et al., 2007), and, in accord, the mouse shows >70% similarity with the coelacanth (lobe-finned fish) sequence (Figure S1). Hence, the ZRS has remained highly invariant against a backdrop of major evolutionary changes to the anatomy of the appendicular skeleton, which includes the transition of fish fins to tetrapod limbs (Gehrke and Shubin, 2016). The structural organization of this class of deeply conserved vertebrate enhancers is under strong selective constraints, and, even in light of binding site redundancies exhibited by transcription factors, few sequence changes are present.

The ZRS is located 800–1,000 kb away from the *Shh* promoter in the mouse and human and is necessary and sufficient for accurately activating and maintaining *Shh* expression in the limb (Lettice et al., 2003, Sagai et al., 2005). An enhancer evolves not simply as a regulator that switches gene expression on or off but must also solve the challenges of regulating expression from a distance (Lettice et al., 2014) while controlling gene activity accurately in space and time and at the appropriate levels. Based on the evolutionary stasis of the ZRS, it is reasonable to expect that the sequence was finely honed during evolution so that that there is little tolerance for sequence change. Indeed, point mutations in and duplications of the ZRS result in a spectrum of appendicular skeletal defects (Anderson et al., 2012). Point mutations in well over 20 different positions scattered across the ZRS cause autosomal dominant limb defects, called “ZRS-associated syndromes” (Wieczorek et al., 2010). Some of the conditions associated with ZRS mutations include preaxial polydactyly type 2, triphalangeal thumb polysyndactyly, syndactyly type 4, and Werner mesomelic syndrome (WMS).

To investigate the structural composition of this highly conserved vertebrate enhancer, we used genome editing technology (Dow, 2015) to target deletions in three regions within the ZRS. Because ZRS activity is limb-specific, the phenotypes were expected to be overt, accessible, and nonlethal. The regions that were targeted contain the 5-bp site responsible for WMS (Anderson et al., 2012), the single mutation responsible for hemimelic extra toes (*Hx*) (Lettice et al., 2008) in the mouse, and a previously identified site for binding the HAND2 transcription factor (Osterwalder et al., 2014). This approach generated an overlapping series of mutations and deletions that scan across 250 bp of the endogenous ZRS. Here we show that the ZRS encodes multiple, diverse functions that contribute to the enhancer activity. Spatial restriction of expression is, in part, controlled by a small repressor domain that confines Shh expression to the posterior limb bud margin. In contrast, large overlapping domains regulate expression levels contingent on the number of HOXD binding sites. In addition, in response to insertion mutations, cryptic, unique phenotypes were generated that revealed the functional plasticity potentially encoded in an enhancer. Mutational analysis, however, also showed that, even though the enhancer is highly conserved, it could still tolerate quite substantial losses of sequence information without causing an abnormal phenotype. We propose a collective model for enhancer composition in which discrete activities and redundant sequences in the ZRS accrue to provide a robust regulatory response during development.

## Results

### ZRS Mutations in the Mouse Mimic WMS

**Figure 1:**
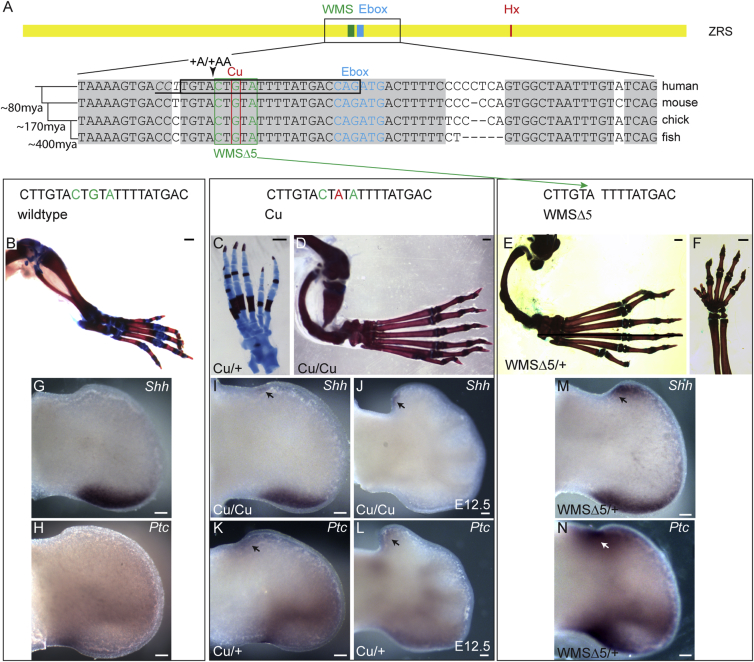
Mutational Analysis of the WMS Site in the ZRS (A) The position of the three sites within the ZRS that were targeted for mutation analysis; the WMS site is boxed. The conservation of the region containing the Werner mesomelic syndrome site is shown. The 3 nucleotides (green) mutated in WMS are shown in a green box and are labeled WMSΔ5, the Cu mutation is highlighted by the red box, and the position of the gRNA is contained in the black box (the PAM site is underlined and in italics). The position of the +A and +AA insertions is also shown. The wild-type and mutant allele sequences are shown at the top. (B, G, and H) The wild-type hind limb (B) and expression patterns of *Shh* (G) and *Ptc* (H) at E11.5 hind limb buds are shown for comparison. (C and D) The hindlimbs of the Cuban mutation (a G > A point change) shows an extra anterior digit in the heterozygote (C), a polydactylous hindlimb, and the hypoplastic tibia in the homozygote (D). (I–L) *Shh* expression in the Cu homozygote at E11.5 (I) and E12.5 (J) and *Ptc* in the heterozygote at E11.5 (K) and E12.5 (L). Ectopic expression is highlighted by the black arrows. (E, F, M, and N) The heterozygous WMSΔ5 deletion mutants. The hindlimb shows the absence of the tibia and polydactyly (E) and, unlike in the Cu mutation, polydactyly on the forelimb (F). Strong ectopic expression of *Shh* (M) and *Ptc* (N) is observed (highlighted by arrows). Scale bars, 500 μm (B and C), 1 mm (D–F), and 100 μm (G–N).

WMS is associated with point mutations in a single, short, 5-bp stretch of the ZRS (green box, Figure 1A) that results in preaxial polydactyly of the hands and feet but is uniquely associated with short limb dwarfism because of tibial hypoplasia. WMS results from any heterozygous point mutation at position 404 (in humans) (Figure 1A, Lettice et al., 2008), a heterozygous A-to-G change two nucleotides downstream at position 406 (Norbnop et al., 2014), and a homozygous C-to-T change at position 402 (VanderMeer et al., 2014; green bases, Figure 1A). These three nucleotide positions lie within a highly conserved site and, to date, are the only sites associated with this syndrome.

Initially, to examine the nature of these mutations in the mouse and to ensure that it is possible to recreate the human abnormality, a G-to-A replacement in position 404 originally reported in a Cuban family (labeled Cu, Figure 1A; Lettice et al., 2003) was generated in mice using conventional “knockin” technology (Lettice et al., 2014). The resulting heterozygous mice exhibited extra preaxial digits on the hindlimbs (Figure 1C), whereas homozygotes, in addition, had bent legs because of tibial dysplasia (Figure 1D). Bone stains confirmed the loss of the terminal portion of each tibia (Figure 1D), which copied the dysplastic tibiae of WMS patients; however, unlike the patients, the forelimbs in mice were unaffected, and tibia dysplasia only occurred in the homozygous mutant.

To further investigate the nature of the dominant mutations at the WMS position, we targeted deletions using CRISPR/Cas9. A guide RNA (gRNA) targeted to this region (black box, Figure 1A) resulted in a number of different deletions and insertions. The most common mutation that was recovered was the precise removal of the five base pairs (called the WMSΔ5 deletion) (green box, Figure 1A) implicated as the site of WMS. The hindlimb phenotype of the WMSΔ5 mutant mice is similar to that observed in the homozygous Cu mutant mice in that the hindlimbs show extra preaxial digits and the tibia is hypoplastic, ranging from partial loss of the distal portion of the bone to its complete absence (Figure 1E). In contrast to the point mutation, these phenotypes occurred in both heterozygous and homozygous WMSΔ5 mice, and both genotypes exhibit preaxial polydactyly (PPD) in the forelimbs (Figure 1F). Thus, the strength of the WMSΔ5 allele is similar to the point mutation in humans. No differences in the severity of the phenotypes were observed between heterozygous and homozygous mice.

Analysis of *Shh* expression in the developing limb buds in the homozygous Cu mutant embryos showed normal expression at embryonic day 10.5 (E10.5), whereas, by E11.5, ectopic anterior expression of *Shh* (Figure 1L) was observed in approximately half of the embryos examined (3 of 7 mice). By E12.5, ectopic *Shh* occurred at the anterior margin (Figure 1J) in an outgrowth of limb tissue in all embryos examined. Heterozygous embryos showed normal *Shh* expression at all stages examined, but analysis of *Ptc1*, a sensitive readout of *Shh* signaling, showed ectopic, anterior expression at both E11.5 and E12.5 (Figures 1K and 1L), showing that low but sufficient levels of ectopic *Shh* were present in all mutant limb buds at these stages. Both heterozygous and homozygous WMSΔ5 mutant embryos showed appreciably more *Shh* and *Ptc* expression at the ectopic site of the hindlimb bud on E11.5 (Figures 1M and 1N) than what was detected in the homozygous Cu embryos, with some also showing ectopic expression in the forelimbs. Thus, the levels of ectopic *Shh* signaling detected reflected the final phenotype, with long-bone abnormalities arising in limb buds expressing higher levels of ectopic *Shh* earlier in development. The clustering of the human mutations within a short 5-bp sequence causing WMS indicates that this is a single important site for transcription factor binding, whereas the deletions confirm that WMS is due to the loss of binding of a repressor that actively represses ectopic expression.

### Small Insertions Extend the Limb Phenotypic Spectrum

**Figure 2:**
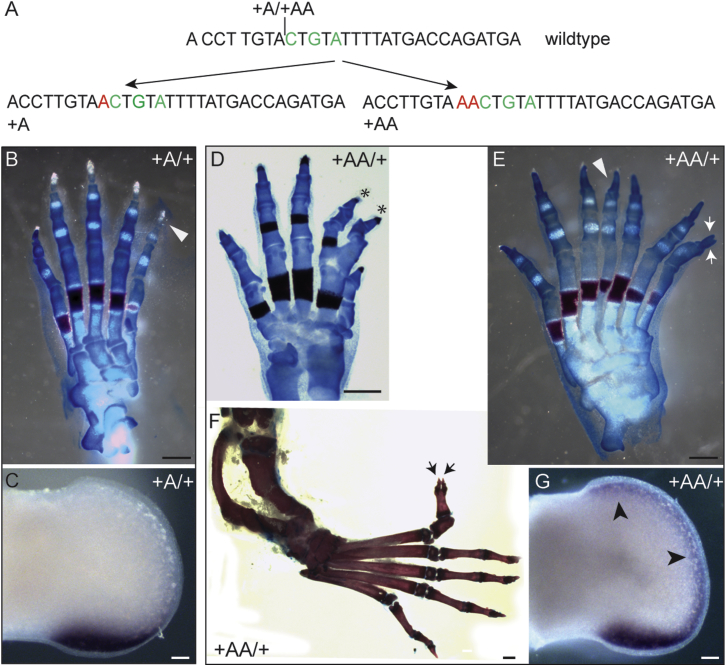
Insertion Mutations Disrupt Limb Development to Generate an Unusual Skeletal Phenotype (A) The position of the adenosine insertions are shown adjacent to the WMS site to create the +A and the +AA mutations. (B) The skeletal features of the +A hindlimb show a triphalangeal digit 1 (arrowhead). (C) Expression of *Shh* on E11.5 in the hindlimb of the +A mutant. (D and E) Forelimb of the +AA mutant (D) showing fusion and duplication of internal digits (asterisks), whereas the hindlimb (E) shows bifurcation at the tip of the extra preaxial digit (arrowheads) and the centrally located extra digit (arrow). (F) Bending of the hindlimb caused by a shortening of the tibia is shown. (G) E11.5 WMS+AA hindlimb bud, showing expression of *Shh* along the entire distal edge (black arrowheads). Scale bars, 500 μm (B, D, and E), 100 μm (C and G), 1 mm (F).

A second set of mutations arose adjacent to the 5-bp WMS site, resulting in the insertion of either one or two additional adenosines (called +A and +AA in Figure 2A). The mutant phenotype generated in the WMS+A mutant heterozygotes was a lengthening of the first digit and, sometimes, the addition of an extra terminal phalange on digit 1 of the hindlimbs (Figure 2B) with normal forelimbs. *Shh* expression appears normal in +A mutant limb buds at E11.5 (Figure 2C); however, the phenotype suggests a low level of expression at the ectopic, anterior margin of the limb bud. Insertion of +AA resulted in a more severe phenotype that has not been previously described for ZRS-associated mutations. This dinucleotide insertion caused typical PPD in the forelimbs (Figure 2D), but, in the hindlimbs, extra digits occurred centrally in the digital array (Figure 2E), occasionally in conjunction with long-bone anomalies (1 in 7 heterozygotes) (Figure 2F). The +AA hind limb buds showed an extended pattern of ectopic expression ranging from the posterior margin all around the distal edge of the limb bud (Figure 2G). The plasticity of a developmental enhancer in producing morphological changes has been investigated in *Drosophila* (Swanson et al., 2010) and in the mouse; it is clear that point mutations in the ZRS give rise to additional preaxial digits and homeotic transformations of the thumb to a finger (Anderson et al., 2012). The +AA mutant embryo presents an unusual skeletal configuration in the digital ray, indicating that further cryptic plasticity is uncovered by mutational events that disrupt the enhancer’s organization.

### Large Regions within the ZRS Are Dispensable

**Figure 3:**
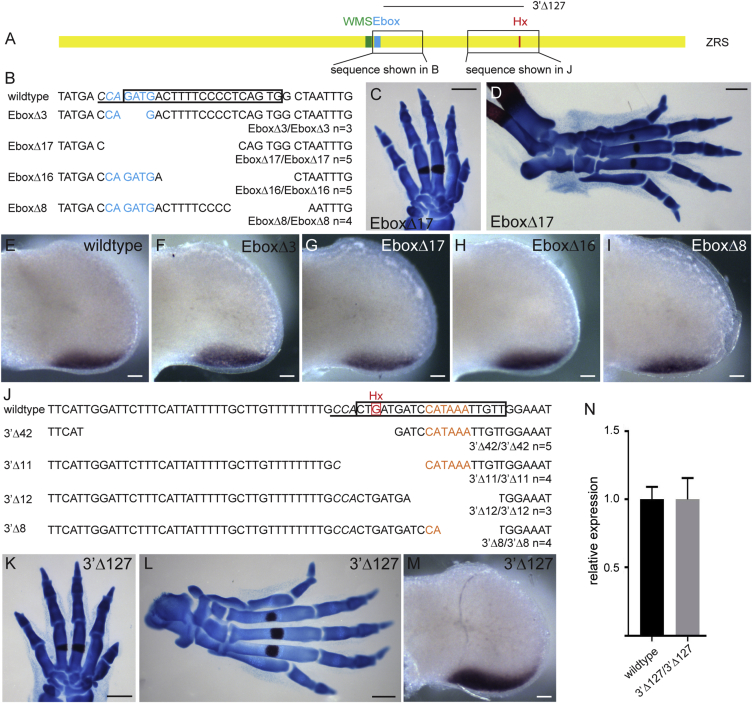
Mutational Analysis of the Ebox and the *Hx* Sites in the ZRS (A) The ZRS (yellow rectangle) and the relative locations of the WMS site, the Ebox, and the Hx mutation are indicated. Boxes highlight the relative positions of the sequences shown in (B) (Ebox) and (J) (Hx), respectively. Linking these two regions, the position of the 3′Δ127 deletion is also shown. The gRNA sequences are boxed in the wild-type sequences in (B) and (J) with the PAM site (italics). (B) The EBox (highlighted in blue font) and the deleted nucleotides for each mutation. The numbers of the homozygous animals analyzed are indicated below each mutation (as n = ). (C and D) Representative forelimb (C) and hindlimb (D) from an EboxΔ17 homozygote demonstrate no detectable deviation from the wild-type. (E–I) *Shh* expression in hindlimbs on E11.5 for the wild-type (E) and EboxΔ3 (F), EboxΔ17 (G), EboxΔ16 (H), and EboxΔ8 (I) homozygotes, showing a normal pattern of expression. (J–L) The mutant sequence affected by the 3′ deletions near Hx (J). The wild-type sequence with the position of the Hx mutation (red base and box) is indicated. The position of Hoxsite 4 is highlighted in orange. The sequences of all the deletions are shown below, and the numbers of the homozygous animals analyzed are indicated below each mutation (as n = ). The apparent unaffected forelimb (K) and hindlimb (L) of the large 3′Δ127deletion are shown. (M and N) The levels of expression of *Shh* at E11.5 hindlimb buds, shown by in situ hybridization (M) and by quantification by qRT-PCR (N) in 3′Δ127 homozygous embryos. Scale bars, 500 μm (C, D, K, and L) and 100 μm (C–I and M). Error bars indicate ± SEM.

We next focused our mutation analysis on two highly conserved regions 3′ of the WMS site (Figure 3A; see Figure S1 for sequence comparison) to delve into the function of previously identified sites that are putatively important for *Shh* gene regulation. Corresponding gRNAs were designed that overlapped these sites (sequences shown as boxes in Figures 3B and 3J, with the protospacer adjacent motif [PAM] sites in italics). One region contains the conserved Ebox that binds the transcription factor HAND2 (Osterwalder et al., 2014; Figure 3A; blue nucleotides, Figure 3B) crucial to the spatial specific activation of *Shh* in the posterior margin of the limb bud. The second region contains the *Hx* mouse point mutation that lies at position 553 (Figure 3A; red nucleotide, Figure 3J), shown by transgenic analysis to operate as a dominant gain-of-function mutation and to encode important structural features crucial for enhancer activity (Lettice et al., 2014). In addition, the *Hx* site is embedded in a large region of the enhancer that is crucial for the long-range activity of the enhancer.

A series of overlapping deletions targeting the Ebox were identified (Figure 3B); two of these disrupted the Ebox: EboxΔ3, which was Ebox-specific, removing the 3 nucleotides from the middle, and EboxΔ17, which deleted the Ebox and surrounding nucleotides. Two other deletions, EboxΔ8 and EboxΔ16, removed nucleotides at the 3′ side of the Ebox. None of these four deletions showed a phenotype either as heterozygotes or homozygotes (the number of homozygotes analyzed is shown as n = in Figure 3B). For example, the largest deletion, EboxΔ17, which disrupts the Ebox and removes surrounding nucleotides, showed wild-type skeletal patterns in both the fore- and hindlimbs (Figures 3C and 3D, respectively). *Shh* expression in the limb buds for all four deletions showed the normal posterior pattern in homozygous embryos (Figures 3F–3I). The EboxΔ3 and EboxΔ17 deletions suggest that removal of a single Ebox site has no detectable effect on *Shh* expression. Possibly, a second conserved Ebox site downstream that has a lower affinity for HAND2 (Osterwalder et al., 2014) may compensate for this loss. The EboxΔ8, EboxΔ16, and EboxΔ17 mutations overlap in a conserved region (Figure 3B; Figure S1), deleting a total of 24 bp. No deletions in this region affected the limb phenotype, showing that a substantial region of conserved information can be disrupted.

Using gRNA targeted to the *Hx* mutation, we identified four deletions, 3′Δ42, 3′Δ11, 3′Δ12, and 3′Δ8, all of which are encompassed in 56 bp, including the Hx site (Figure 3J), and none of these had an effect on limb phenotype. Similar to the deletions created for the Ebox, these removed highly conserved nucleotide stretches: the 3′Δ8 and Δ12 deletion disrupting a HOXD binding site (Hoxsite 4; orange nucleotides, Figure 3J; Figure 5) (see below). 3′Δ11 and 3′Δ42 remove the *Hx* mutant site, and no polydactylous phenotype is detected in the heterozygotes, confirming that, unlike the WMS mutations, the *Hx* point change is a gain-of-function mutation (Lettice et al., 2014). The two other deletions, 3′Δ8 and 3′Δ12, do not contain the Hx mutant site but do remove the adjacent, highly conserved sequences containing the Hoxsite4, and these do not show a heterozygous phenotype. Homozygous mutants were made for all of these deletions, and no phenotype was detected (numbers are shown in Figure 3J). The larger, 127-bp deletion (3′Δ127) (Figure 3A; Figure S1) confirmed this tolerance for loss of conserved sequence. The large 3′Δ127 allele showed no dominant effect on digit number, and, in the homozygous state, there was no influence on the limb phenotype (n = 7) (Figures 3K and 3L), whereas both in situ hybridization and qRT-PCR showed no appreciable change in the expression profile or levels (Figures 3M and 3N). This deletion showed that a large region of conserved sequence can be deleted from this enhancer. Because the mutational analysis was performed at the endogenous locus, the lack of a phenotype suggests that the loss of the 3′Δ127 sequence is compensated for, thus indicating that there is encoded redundancy within the enhancer.

#### Large Deletions Encompassing the WMS Site Incrementally Affect Expression Levels

**Figure 4:**
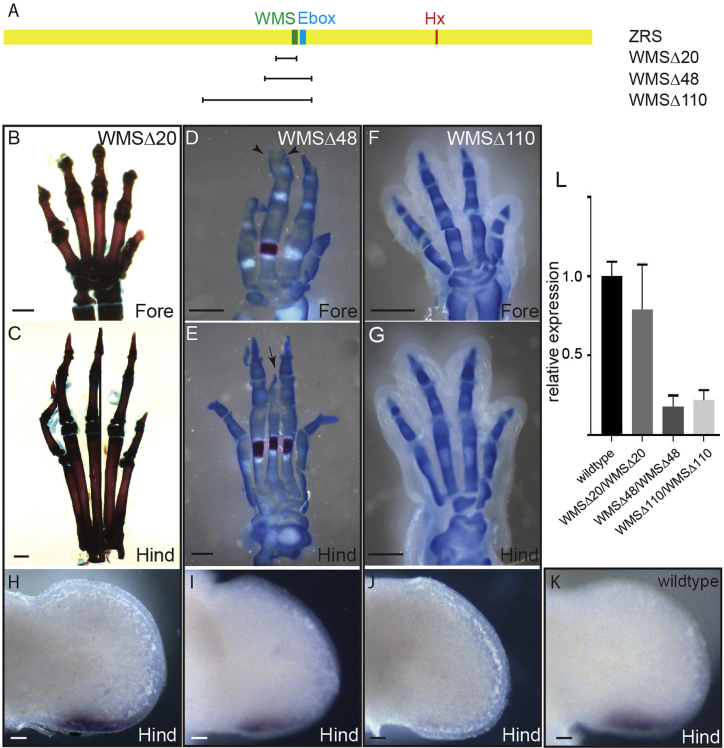
Deletions Near the WMS Site Reduce the Levels of *Shh* Expression (A) The three deletions WMSΔ20, WMSΔ48, and WMSΔ110 are shown relative to the position of the WMS (green line), Ebox sites (blue line), and Hx mutation (red) within the ZRS (yellow rectangle). (B, C, and H) The homozygous WMSΔ20 mutants. No limb abnormalities are detected in the forelimb (B) or the hind limb (C) of the homozygous WMSΔ48. (D–G) WMSΔ48 limbs (D) and (E). The forelimb (D) shows loss of a digit and two terminal phalanges on the adjacent digit (arrowheads), and the hindlimb in (E) shows partial loss of digit 3 (arrow). Loss of the middle digit in the forelimb (F) and hind limb (G) are shown in the WMSΔ110 deletion. (H–K) *Shh* expression on E11.5 in the hindlimbs of WMSΔ20 (H), WMSΔ48 (I), WMSΔ110 (J), and wild-type (K) embryos. (L) The outcome of the quantification by qRT-PCR of *Shh* expression in E11.5 limb buds from wild-type and WMSΔ20, WMSΔ48, and WMSΔ110 homozygous embryos. WMSΔ48 and WMS110 expression levels are significantly (p < 0.001) lower than wild-type levels (Kruskal-Wallis test with Dunn’s multiple comparisons). Error bars indicate ± SEM. Scale bars, 1 mm (B and C), 500 μm (D–G), and 100 μm (H–K).

Three other deletions were generated (Figure 4A; Figure S1) when making the WMS mutations: a 20-bp deletion, WMSΔ20, which included the 5-bp site of the WMSΔ5 and two other deletions, WMSΔ48 and WMSΔ110, both of which lost 21 bp on the 3′ side of the WMS site, removing the Ebox element but extending to different positions at the 5′ end. The WMSΔ20 deletion, unexpectedly, showed no observable limb phenotype, neither a dominant phenotype displaying extra toes nor, in the WMSΔ20/WMSΔ20 homozygote, a loss of activity phenotype displaying skeletal deficiencies (n = 5) (Figures 4B and 4C). The WMSΔ20 mouse was further crossed to the *Shh*-null mutation to make the WMSΔ20/*Shh*^*null*^ compound heterozygote to expose any subtle loss of activity, but these, again, showed no abnormal phenotype (n = 5). Analysis of *Shh* expression in WMSΔ20 homozygotes showed little observable differences in expression pattern (Figure 4H) compared with the wild-type (Figure 4K), and levels of expression measured by qRT-PCR were not affected significantly (Figure 4L). Thus, the deleterious phenotypic effects of the WMSΔ5 mutations were lost in the larger WMSΔ20 deletion.

Two deletions, WMSΔ48 and WMSΔ110 (Figure 4A), were examined, and, in the homozygote, removal of these sequences resulted in loss of digits. The WMSΔ48 mutation showed loss of up to one digit on each on the forepaws (Figure 4D), with some elements being retained and soft and hard tissue syndactyly and fusion being observed. The hindpaws were mildly affected, some showing only partial loss of a single digit (digit 3) (Figure 4E). The WMSΔ110 embryos showed a precise loss of one digit on all four paws (Figures 4F and 4G), with the rest of the digits apparently unaffected. Because these phenotypes were seen only in the homozygous state, these were loss-of-activity mutations resulting in a decrease in enhancer activity. Indeed, *Shh* expression was lower but was retained at the posterior margin of the limb bud at E11.5, but by in situ hybridization, levels in Δ48 (Figure 4I) appeared to be appreciably lower than in the wild-type (Figure 4K), with further reductions in WMSΔ110 (Figure 4J). Levels of RNA measured by qRT-PCR showed a significant reduction in *Shh*, in both WMSΔ110 and WMSΔ48, compared with the wild-type (Figure 4L).

### Multiple Conserved HOX Binding Sites Control the Expression Levels of *Shh*

**Figure 5:**
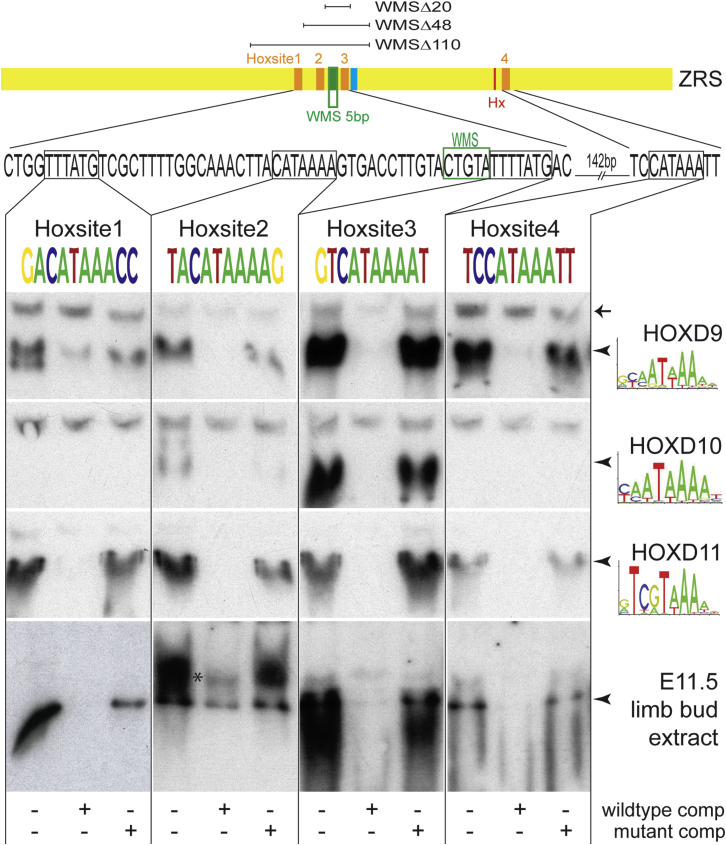
HoxD Binding to Conserved Motifs in the ZRS The top line shows the genomic sequence around the 4 Hox binding sites (designated Hoxsites 1–4), with the position of the WMS region indicated. The relative positions of the WMSΔ20, WMSΔ48, and WMSΔ110 deletions are indicated. Below, the sequences of the 4 Hoxsites are shown in the same orientation. The consensus binding sites for each of the proteins (HOXD9, D10, and D11) are shown as position weight matrices under their gene names. For each triplet of EMSAs, the lanes are shown as binding to a labeled Hoxsite oligonucleotide with no competition, with excess of the wild-type oligo as competitor and with the mutated Hoxsite oligo in competition to show specificity of binding to the Hoxsite. Specific binding is indicated by the arrowheads. In the case of the E11.5 limb bud extract binding to Hoxsite 2, a higher mobility shift is observed, indicated by the asterisk. The non-specific band (arrow) is marked as a comparison with Figure S2.

Within the ZRS, a highly conserved 6-bp element composed of the sequence CATAAA was detected at four positions (boxed in Figure 5 and Figure S1). This 6-bp sequence is embedded in sites that compare well with the consensus motif established for the 5′ *Hoxd* genes (the motif for HOXD9–11 is shown in Figure 5), and these were numbered Hoxsites 1–4. Genetic analysis of the HOX complexes previously demonstrated that the 5′ *Hoxd* genes (*Hoxd*10–13) and that their counterparts in the *Hoxa* locus regulate *Shh* expression in the limb (Tarchini et al., 2006), and chromatin immunoprecipitation (ChIP) showed that at least two Hox proteins, HOXD10 and 13, directly bind to the ZRS (Capellini et al., 2006). Three of these identified sites (Hoxsites 1, 2, and 3) are contained within the Δ110 deletion, the Δ48 contained two sites (Hoxsites 2 and 3), and the Δ20 contained only Hoxsite 3 (Figure 5; Figure S1). Hoxsite 4 was deleted in the series that included the *Hx* site (Figure 3J) discussed above. Each of the 5′ *Hoxd* genes was cloned into the vector pT7CFE1-CHis for subsequent expression in the human in vitro expression system (1-Step Human Coupled IVT Kit, Thermo Fisher Scientific). The 5′ HOXD proteins were synthesized (Figure S2) and used in an electromobility shift assay (EMSA) to establish binding to double-stranded oligonucleotides containing one of these four sites (Table S1). The in vitro-synthesized HOXD9, 10, and 11 proteins showed the highest binding activity with these sites (Figure 5), whereas HOXD12 and 13 showed lower activity across all oligos (Figure S2B). The HOXD proteins showed different preferences for these sites; HOXD9 and 11 bound all four sites, with D9 showing a preference for sites 3 and 4, whereas D11 favored Hoxsites 1, 2, and 3. HOXD10 bound site 3 but bound weakly to Hoxsites 1, 2, and 4 (Figure 5). Specificity of binding was shown using competitor oligonucleotides with either wild-type sequence or Hox binding site mutations (Figure 5; Table S1). Nuclear extracts from E11.5 limb buds were also used in the EMSA (Figure 5) and were found to bind to all sites, and the binding was specific for the putative HOX binding motif.

**Figure 6:**
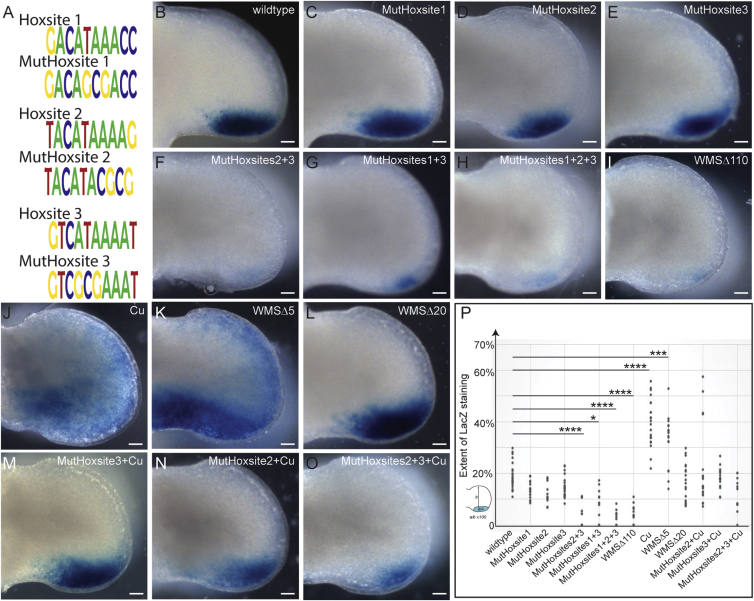
Transgenic Analysis of Embryos Carrying Mutant ZRS Sequences (A) The sequences of the wild-type Hoxsites 1–3 and the mutated sequences (designated MutHoxsite) that were used in the transgenic constructs. (B–E) Limb buds from transgenic embryos (E11.5) carrying the following ZRS sequences driving LacZ expression: the wild-type ZRS sequences (B); MutHoxsite 1 (C), MutHoxsite 2 (D), and MutHoxsite 3 (E). (C)–(E) show no effect on expression of mutating single Hoxsites. (F–H) Mutating combinations of sites results in lower LacZ expression: MutHoxsite2+3 (F), MutHoxsite 1+3 (G), and MutHoxsite 1,2+3 (H). (I) The low level of expression in MutHoxsite 1,2+3 is reproduced in the WMSΔ110 construct. (J–L) Addition of the Cu point mutation (J) or deletion of WMSΔ5 (K) results in distal and ectopic anterior expression, whereas deletion of WMSΔ20 (L) returns expression to wild-type levels. (M–O) The Cu mutation in combination with mutant Hox sites: MutHoxsite 3+Cu (M), MutHoxsite 2+Cu (N), and MutHoxsite 2+3+Cu (O). (P) Graphical representation of the LacZ expression patterns resulting from mutations within the ZRS. The width of the expression domain was divided by the width of the limb and expressed as a percentage. One spot represents the extent of reporter gene (*LacZ*) expression for each individual limb from a set of transient transgenic embryos. Data were subjected to one-way ANOVA and Tukey HSD test, and those that differ significantly from the wild-type are indicted (^∗^p ≤ 0.05, ^∗∗∗^p ≤ 0.001, ^∗∗∗∗^p ≤ 0.0001). Scale bars, 100 μm.

The role played by the three HOX sites (Hoxsites 1–3) contained in the Δ110 deletion on ZRS activity was assayed in a series of transgenic embryos. Each site was mutated by replacing three bases in the CATAAA element (the mutations for each site are shown in Figure 6A) in a construct carrying the mutated, full-length ZRS driving the expression of a *LacZ* reporter gene. Mutations in each of these HOXD binding sites were made individually or in combination, and expression was examined on E11.5 in each injected transgenic embryo (the transient G_0_ embryo). As a measure of the relative extent of expression in each transgenic embryo, the width of expression as a percentage of limb bud width was plotted to show the trends (individual limbs are represented by dots in Figure 6P). Mutations in individual Hoxsites had no observable effects on transgenic expression in the limb bud (compare Figure 6B with Figures 6C–6E; Figure 5P); however, mutations in the two sites (Hoxsites 2 and 3) that were contained in the Δ48 deletion or mutations in Hoxsites 1 and 3 showed detectably decreased expression (Figures 6F, 6G, and 6P). Mutation of all three sites (Hoxsites 1-–3) showed even further decreases (Figures 6H and 6P) comparable with the ZRS carrying the Δ110 deletion (Figures 6I and 6P). The accumulative decrease in expression of the endogenous *Shh* in the Δ48 and Δ110 deletions correlates with the progressive loss of the HOXD binding sites Hoxsites 1–3.

### Deletions of the WMS Domain Restore the Wild-Type Phenotype

The deletion in WMSΔ20 removes the WMS repressor site but also includes Hoxsite 3. Transgenics carrying either the Cu point mutation (Lettice et al., 2003; Figure 6J) or the WMSΔ5 deletion (Figure 6K) drives reporter gene expression to an elevated level in the posterior margin of the limb bud (Figure 6P) with appreciable ectopic expression. The WMSΔ20 deletion appears to return transgenic expression to wild-type levels (Figures 6L and 6P). Loss of the WMS repressor in combination with the mutant Hoxsite 3 binding site may be sufficient to nullify the increased and ectopic expression by the WMS mutations. To examine this possibility, transgenic mice carrying the Cu mutation in the presence of the 3-bp replacement (see above) that disrupts Hoxsite 3 was used in the transgenic assay and showed no detectable upregulation of the reporter on E11.5 and, importantly, no ectopic expression (Figures 6M and 6P). To show that the lack of ZRS upregulation was due to the independent action of the WMS mutations and loss of Hoxsite binding, transgenics carrying the Cu change and a different Hoxsite mutation (Hoxsite 2) also predominantly showed the wild-type pattern of expression (one of five G_0_ embryos retained ectopic expression) (Figures 6N and 6P). Reductions in expression were shown in the presence of the WMS point mutation when two Hoxsites were mutated (Figures 6O and 6P). The transgenic expression reflects the WMSΔ20 deletion, suggesting that when the WMS mutation, which affects the binding of a repressor, is in the presence of mutations that disrupt binding of an activator, the mutations effectively cancel each other out, giving rise to wild-type expression levels. The independent action of these opposing activities emphasizes the combinatorial nature of elements that operate in the ZRS.

## Discussion

**Figure 7:**
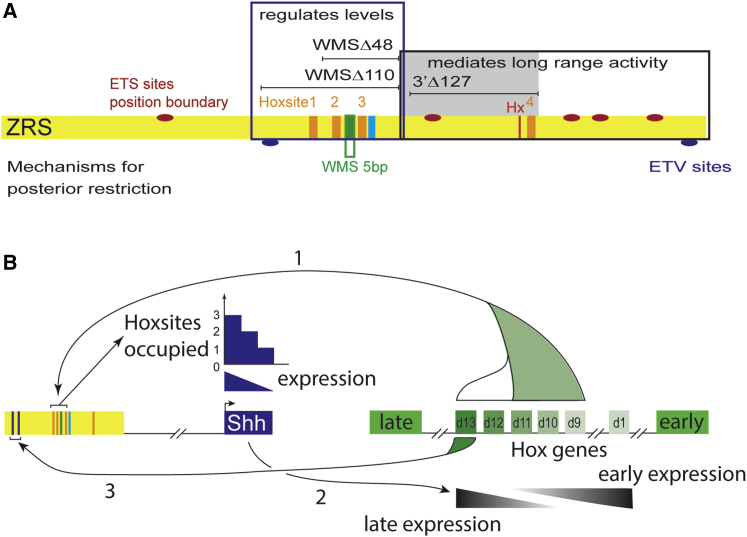
Schematic of the Composition of the ZRS and Its Role in the *Shh* and HoxD Regulatory Loop (A) A representation of different functional regions and sites established for the ZRS. The ZRS is represented by the yellow rectangle, and the positions of the WMS 5bp site (green), the Ebox (blue), the Hx mutations (red), and Hoxsites (orange) are indicated in the ZRS. The positions of the 5 ETS sites that control the position of the expression boundary are represented by the red ovals. The region that contributes to regulating levels is in the blue box, and the deletions that revealed this activity are shown. The region that mediates long-range activity is shown in the black box. The large region of this domain that is redundant is shown by the gray shading. The two systems that control posterior restriction are shown below the ZRS rectangle, indicating the position of the two ETV binding sites and the position of the WMS 5-bp site. (B) Summary of the positive feedback loop between the 5′ *HoxD* genes and *Shh* to reinforce expression of *Shh.* The ZRS (yellow box) and its position relative to *Shh* is shown on the left, whereas a schematic of the HoxD complex, including the two flanking regulatory domains (the early enhancer and the late enhancer), is depicted by green boxes on the right. Early-expressing 5′ HOXD proteins bind Hoxsites 1–3 within the ZRS to establish the levels of *Shh* expression in the initial stages of limb development (arrow 1). The levels of Shh expression are dependent on the number of Hox sites occupied. SHH, in turn, is crucial for the shift in HoxD gene expression to the later genes, in particular *Hoxd13* (arrow 2). HOXD13 subsequently binds to sites at the 5′ end of the ZRS, as established by Leal and Cohn (2016) (arrow 3), to maintain *Shh* expression later in limb development.

The aim of this study was to investigate the composition of a vertebrate enhancer that falls into the highly conserved class of elements (Ovcharenko et al., 2004). These vertebrate enhancers represent a class in which the structural architecture is under selective constraints, resulting in apparent structural inflexibility in both the redundancy and the positioning of transcription factor binding motifs. These enhancers, which range in size from 100 bp to >1 kb, have the capacity to bind a substantial number of transcription factors, arguing that, within a single functional element, there is also a degree of structural complexity. This structural complexity has enabled the dissection of the ZRS into discrete regulatory activities. The expression pattern of the *Shh* gene in the limb bud is a consolidation of activities that control restriction of expression to the posterior margin, spatial and temporal expression, levels of expression, and long-range promoter activation (summarized in Figure 7A).

Other examples of a complex arrangement of components have been reported, including an elegant analysis of the *Drosophila spa* enhancer that showed that structural organization underlies correct developmental gene expression (Swanson et al., 2010); for instance, sequence elements were defined that regulate long-range activity and others that repress expression in the wrong cell type. Our analysis surveying deletions further showed a complex organization that included unexpected redundancy incorporated into the enhancer. The model for enhancer action we propose here is one that relies on consolidation of discrete, discernible activities acting as a collective. This collective model suggests an integration of these discrete activities and redundant elements in delivering robust spatiotemporal developmental expression.

### Hox Genes Function at the ZRS to Regulate Levels of Expression

We show the homotypic clustering of conserved HOXD binding sites (Hoxsites 1–4) in the ZRS. At least three of these sites (Hoxsites 1–3) are clustered in a 110-bp domain of the ZRS and regulate levels of *Shh* expression. The 5′ *Hoxd* genes, which include *Hoxd 9–13*, are fundamental to limb patterning and are expressed in a temporal collinear fashion, with the *Hoxd9* gene expressing earliest in the limb bud, followed in sequence by *Hoxd13* being expressed latest (Tarchini and Duboule, 2006). A clustering of highly conserved sites that contain the core motif for binding the 5′ HOXD proteins operates in an accumulative manner to regulate the activity levels of the ZRS enhancer. In an in vitro assay, we showed that the early 5′ HOXD proteins (HOXD9–11) bind this motif, suggesting that these play an initial role in establishing the activity levels of the ZRS. The region of the ZRS that contains three of these HOXD motifs is crucial for activity, and deletions show decreasing *Shh* expression corresponding to the number of Hox binding sites lost. In addition, loss of a Hox binding site counterbalances the increased and ectopic expression generated by the loss of the WMS repressor site. Thus, multiple HOXD factors coordinate, through binding at multiple sites, the expression levels of *Shh*.

Additional HOXD binding sites have been identified near the 5′ end of the ZRS that have a preference for binding HOXD13 (Leal and Cohn, 2016; Figure 7B). Two sets of HOX sites, therefore, regulate gene expression, reacting to the temporal changes in the expression of the 5′ *Hoxd* genes. We suggest that the sites we identified play a role in establishing the levels of *Shh* expression in the initial stages of limb development by binding the early-expressing 5′ HOXD proteins (HOXD10 and 11) but adjust to the changing embryonic environment within the developing limb by also interacting with the later-expressed HOXD13 at different sites.

This establishes a regulatory loop that operates by positive feedback, reinforcing the expression of the *Shh* gene by the 5′ *Hoxd* genes (Figure 7B). The early 5′ HOXD proteins interact with the ZRS at the HOX binding sites examined in this study to establish the levels of Shh expression based on the sum of the sites occupied (arrow 1, Figure 7B). SHH, in turn, is crucial for the shift in the regulation of *HoxD* gene expression from a set of early-acting enhancers to the enhancers at the 5′ end of the gene cluster (arrow 2, Figure 7B) that regulate the late-expressing genes, in particular *Hoxd13* (Zákány et al., 2004). HOXD13 subsequently binds to sites at the 5′ end of the ZRS, as established by Leal and Cohn (2016) (arrow 3, Figure 7B). We suggest that a temporal response to *HoxD* genes is important for continued *Shh* expression as the regulatory environment in the limb bud changes over the 2 days when *Shh* is expressed in the mouse limb.

In accord, python and boa snakes, which have lost the HOXD13 binding sites in the ZRS, initially express *Shh* in the rudimentary limb buds, presumably dependent on the early 5′ HOXD protein binding sites we established; however, *Shh* expression is lost later, and limb development is prematurely terminated. This loss of the HOXD13 binding sites, in combination with loss of an ETS binding site (Leal and Cohn, 2016, Kvon et al., 2016), is responsible for the loss of limbs in these snakes.

Homotypic clustering of binding sites in the ZRS appears to play a number of roles in determining the spatial expression pattern of *Shh* expression in the embryonic limb bud. We previously showed multiple binding sites for the ETS factors, ETS1 and GABPα (Lettice et al., 2012). Multiple occupancy of these sites determines the extent of the boundary of *Shh* expression. Mutations in the human ZRS that generate an extra ETS site result in the extension of this expression boundary and ectopic expression in the limb bud, resulting in preaxial polydactyly (Lettice et al., 2012, Laurell et al., 2012). Here, occupancy of multiple HOXD binding sites regulates the levels of expression, and sequential loss of these sites results in a gradual decrease in expression levels. Homotypic clustering of binding sites in the ZRS, therefore, operates to incrementally adjust expression of the *Shh* gene and is therefore a fundamental mechanism for fine-tuning the regulatory activity of the enhancer.

### ZRS Activity and Congenital Abnormalities

Mutations in the human ZRS cause skeletal abnormalities (Anderson et al., 2012). The point mutations act in a dominant fashion to cause digital abnormalities, and, presumably, most operate by switching restricted posterior expression to expression at both the posterior margin and an ectopic site at the anterior margin. One set of point mutations generates additional binding sites for ETS1/GABPα transcription factors, acting as dominant gain-of-activity mutations (Lettice et al., 2012). WMS, on the other hand, is highlighted by point mutations in three distinct positions in a single 5-bp site. The action of these point mutations, confirmed by the WMSΔ5 deletion data, is consistent with loss of binding of a repressor and, thus, an overall loss of functional activity. Hence, point mutations in the ZRS have two modes of action, operating as both gain and loss of activity, but both result in dominant genetic effects on the phenotype.

### Insertions Reveal Cryptic Phenotypes

The WMS+AA insertional mutation reveals an unusual phenotype, showing the latent capacity for phenotypic innovation carried by this enhancer. The potential for appreciable morphological change shows that developmental enhancers may have the capacity for change without undergoing large sequence and structural changes in evolution. Selection against such substantial morphological changes may be one of the evolutionary constraints operating on the ZRS, but, in contrast, this also highlights the capacity for appreciable change in vertebrate evolution. These additions reveal the plasticity that is potentially hidden within an enhancer in controlling the phenotype and highlights mechanisms that may be available for phenotypic change during the evolution of an enhancer.

### Evolution of the ZRS

The function of a *cis* regulator is encoded in its molecular architecture. Overlapping deletions in the ZRS that would predictably disrupt this architecture were made near and encompassing the proposed Ebox binding site and the WMS site that removed a total of 44 bp of highly conserved sequence, and these do not affect the limb phenotype. Moreover, the large 3′Δ127 mutation removes the conserved sequence from the 3′ half of the ZRS, which overlaps these 44 bp and also displays neither a limb phenotype nor a detectable reduction in expression. The ability to compensate for loss of sequence information suggests that there is encoded redundancy within the enhancer. This seemingly redundant activity may contribute to phenotypic robustness during development. Robustness is deemed important to buffer developmental processes from environmental and genetic perturbations and was proposed as canalization by Waddington (1942). For enhancers, such redundancy is widespread in *Drosophila* (Cannavò et al., 2016). Secondary or “shadow enhancers” in *Drosophila* provide redundant activity for the primary enhancer, and analyses of specific examples show that these can buffer a developmental process against environmental perturbations (Frankel et al., 2010, Perry et al., 2010). It is clear that the ZRS is able to tolerate losses of a highly conserved sequence without affecting the phenotype under ideal breeding conditions and a defined genetic background. In contrast with shadow enhancers, the robustness apparent in the ZRS is encoded within a single enhancer element because no compensatory activity is apparent in ZRS deletions. Hence, redundancy is an important characteristic of enhancers, whether this is encoded in secondary enhancers or contained within a single element, such as in the ZRS.

The evolutionary stability of the ZRS sequence raises a number of questions about the evolvability of this and perhaps other highly conserved enhancers. In addition, this stability occurs in light of the major morphological changes that have occurred to the limb during vertebrate evolution. Thus, the ZRS displays low sequence variability in a morphologically plastic developmental system. The recurrent role the ZRS plays in the diverse species analyzed so far is to ensure that *Shh* is expressed specifically along the posterior margin of the developing appendage, whether it is an embryonic fin (Dahn et al., 2007) or a limb bud. Many of the genes and signaling pathways known to regulate *Shh* in the mouse, such as the *HoxD* complex, *Hand2*, *Gli3*, and the fibroblast growth factor (FGF) pathway, are implicated in chick and fish, suggesting that the gene network responsible for *Shh* activation is also conserved (Gehrke and Shubin, 2016). For vertebrate enhancers, which are found in all vertebrate classes from cold-blooded fish to warm-blooded mammals, it is unlikely that the apparent robustness is a response to environmental factors because these insults would be different for each species. The genetic network of transcription factors and signaling pathways that converge at the ZRS is complex, and we suggest that the regulatory robustness observed for the ZRS buffers against variability and perturbations in this genetic network. This network, which converges at the ZRS, would therefore have evolved early in vertebrates, operating relatively unchanged in the appendicular skeleton in all classes of vertebrates. The conserved enhancer architecture is a response to this complex network and would be a constant factor that pervades species evolution during the morphological changes that have occurred during the fin-to-limb transition.

## Experimental Procedures

### Production and Analysis of CRISPR Mice

gRNAs were designed using the Optimized CRISPR design site (http://crispr.mit.edu/), and the exact guides were chosen on the basis of their precise location relative to the desired sites in the ZRS (The selected oligonucleotides are listed in Table S1). Oligos were cloned into the px330 vector (Addgene) (Cong et al., 2013), and DNA was prepared using the QIAGEN Plasmid Maxi kit according to the manufacturer’s protocol.

Transgenic mice were made by pronuclear injection of plasmid DNA at a concentration of 5 ng/μL. All resulting pups were screened phenotypically and had their ZRS sequence amplified by PCR and sequenced. All genotyping was performed by direct sequencing.

Skeletal preparations were stained simultaneously with alizarin red and Alcian blue (Nagy et al., 2009a, Nagy et al., 2009b). Whole-mount in situ hybridization was performed as described previously (Hecksher-Sørensen et al., 1998) using probes for *Shh* (Echelard et al., 1993) (a kind gift from Andy McMahon) and *Ptc* (Hayes et al., 1998) (a kind gift from Chris Hayes). qRT-PCR for Shh expression was performed on individual pairs of limb buds as described by Lettice et al. (2014). Expression was normalized within a litter to the wild-type level, and statistical significance was calculated by Prism using Kruskal-Wallis test with Dunn’s multiple comparisons. Mouse studies were approved by the University of Edinburgh animal welfare and ethical review board (AWERB) and carried out under the auspices of the United Kingdom Home Office.

### EMSAs/In Vitro Translated Proteins

The coding regions of mouse *Hoxd 9–13* were amplified by PCR using KOD polymerase (Merck Millipore). The primers used are listed in Table S1. Products were cloned into the expression vector pT7CFE1-CHis for subsequent expression in the human in vitro expression system (1-Step Human Coupled IVT Kit, Thermo Fisher Scientific) following the manufacturer’s instructions. Synthesis of each of the HOXD proteins was verified on a western blot using a rabbit anti-His tag antibody (2365, Cell Signaling Technology) (Figure S2) before the protein was used in an EMSA. The double-stranded oligonucleotides were biotin-labeled by the manufacturer (Sigma) and assayed to ensure that each was labeled to a similar specific activity. EMSAs were conducted as described previously (Lettice et al., 2012), and we used either 2 μL of a 1/25 dilution of protein from the in vitro translated (IVT) reaction or 4 μg of limb bud extract (prepared using the NE-PER Nuclear and Cytoplasmic Extraction Reagent Kit, Thermo Scientific). The specificity of binding was confirmed by competition with 100× excess of either unlabeled wild-type or mutant (mut) Hoxsite oligonucleotides. (Table S1).

### Mutant ZRS Transgenic Constructs

Reporter gene transgenic analyses were made as described previously (Lettice et al., 2012). The mutant ZRS deletions (WMS Δ5, Δ20, and Δ110) used were generated by PCR using primers ZRSF and R (Table S1) from the appropriate mutant DNA. The Cu point mutation and MutHoxsite constructs were created using the primers listed in Table S1 and the QuikChange II Site-Directed Mutagenesis Kit (Agilent). For combinations of sites, multiple rounds of mutagenesis were conducted, and the correctly mutated ZRS was subsequently cloned into fresh lacZ-containing vector.

## Author Contributions

Conceptualization, L.A.L.; Methodology, L.A.L.; Validation, L.A.L.; Investigation, L.A.L., P.D., and C.D.A.; Writing – Original Draft, R.E.H.; Writing – Review & Editing, L.A.L. and R.E.H.; Visualization, L.A.L.; Supervision, L.A.L. and R.E.H.; Funding Acquisition, R.E.H.

## Supplemental Information

Document S1. Figures S1 and S2 and Table S1

Document S2. Article plus Supplemental Information
