# Supplementary material for: The Conserved Sonic Hedgehog Limb Enhancer Consists of Discrete Functional Elements that Regulate Precise Spatial Expression
Source: Cell Rep. 2017 Aug 8;20(6):1396–408. doi: 10.1016/j.celrep.2017.07.037 (PMC5561167; doi:10.1016/j.celrep.2017.07.037)
Supplement: Document S1. Figures S1 and S2 and Table S1 [file mmc1.pdf]

Cell Reports, Volume *20*

Supplemental Information

The Conserved Sonic Hedgehog Limb Enhancer Consists of Discrete Functional Elements that Regulate Precise Spatial Expression

Laura A. Lettice, Paul Devenney, Carlo De Angelis, and Robert E. Hill

Table S1- Relates to Figures 5 and 6
Oligonucleotides used.

**Px330 oligos**	
WMS guideF	CACCGTGGTCATAAAATACAGTACA
WMS guideR	AAACTGTACTGTATTTTATGTCCAC
EBox guideF	CACCGCACTGAGGGGAAAAGTCATC
EBox guideF	AAACGATGACTTTTCCCCTCAGTGC
3' guideF	CACCGAACAATTTATGGATCATCAG
3' guideR	AAACCTGATGATCCATAAATTGTTC
**Hox gene IVT constructs**	
HoxD9F	CATGAT**CATATG**TCGTCCAGTGGCACCC
HoxD9R	CATGAT**CTCGAG**GTCTCCTTTAGGGCACTTCTC
HoxD10F	CATGAT**CATATG**TCCTTTCCCAACAGCTCTC
HoxD10R	CATGAT**CTCGAG**AGAAAAGGTGAGGTTGGCGGTC
HoxD11F	CATGAT**CATATG**AACGACTTTGACGAGTGCG
HoxD11R	CATGAT**CTCGAG**AAATAAGGGGTTTCCAGTGAAATATTG
HoxD12F	CATGAT**CATATG**TGTGAGCGCAGTCTCTAC
HoxD12R	CATGAT**CTCGAG**ATAGAGGGCCAGTGCTTGCTC
HoxD13F	CATGAT**CATATG**AGCCGCTCGGGACTTGG
HoxD13R	CATGAT**CTCGAG**GGAGACAGTGTCTTTGAGCTTG
**Hoxsites EMSA oligos**	
Hoxsite 1F	TTGTCCTGG**TTTATG**TCGCTTTTG
Hoxsite 1R	CAAAAGCGA**CATAAA**CCAGGACAA
MutHoxsite 1F	TTGTCCTGG**TTgcgG**TCGCTTTTG
MutHoxsite 1R	CAAAAGCGA**CcgcAA**CCAGGACAA
Hoxsite 2F	CAAACTTA**CATAAAA**GTGACCTTGT
Hoxsite 2R	ACAAGGTCAC**TTTTATG**TAAGTTTG
MutHoxsite 2F	CAAACTTA**CATAcgc**GTGACCTTGT
MutHoxsite 2R	ACAAGGTCAC**gcgTATG**TAAGTTTG
Hoxsite 3F	TGTACTGTAT**TTTATG**ACCAGATGACT
Hoxsite 3R	AGTCATCTGGT**CATAAAA**TACAGTACA
MutHoxsite 3F	TGTACTGTA**TTTcgcG**ACCAGATGACT
MutHoxsite 3R	AGTCATCTGGT**CgcgAAA**TACAGTACA
Hoxsite 4F	CTGATGATC**CATAAA**TTGTTGGAA
Hoxsite 4R	TTCCAACAA**TTTATG**GATCATCAG
MutHoxsite 4F	CTGATGATC**CcgcAA**TTGTTGGAA
MutHoxsite 4R	TTCCAACAA**TTgcgG**GATCATCAG
**LacZ Transgenic constructs**	
ZRS F	GATCAT**AAGCTT**TACTTTAAGCCATCTTTG
ZRS R	GATCAT**AAGCTT**CACATAGAACACTTAGTGAG
Mutate ZRS oligos	
Cu F	GACCTTGTACTaTA**TTTTATG**ACCAGATGACTTTTCCCTC
Cu R	GAGGGAAAGTCATCTGGT**CATAAAA**TAtAGTACAAGGTC
MutHoxsite 1F	CAGTTTGAGATTGTCCTGG**TcgcTG**TCGCTTTTGGCAAAC
MutHoxsite 1R	GTTTGCCAAAAGCGA**CAgcgA**CCAGGACAATCTCAAACTG
MutHoxsite 2F	GTCGCTTTTGGCAAACTTA**CATAcgc**GTGACCTTGTACTG
MutHoxsite 2R	CAGTACAAGGTCAC**gcgTATG**TAAGTTTGCCAAAGCGAC
MutHoxsite 3F	GACCTTGTACTGTA**TTTcgcG**ACCAGATGACTTTTCCCTC
MutHoxsite 3R	GAGGGAAAGTCATCTGGT**CgcgAAA**TACAGTACAAGGTC
MutHoxsite 3F+Cu	GACCTTGTACTaTA**TTTcgcG**ACCAGATGACTTTTCCCTC
MutHoxsite 3R+Cu	GAGGGAAAGTCATCTGGT**CgcgAAA**TAtAGTACAAGGTC

Figure S1-relates to Figure 1

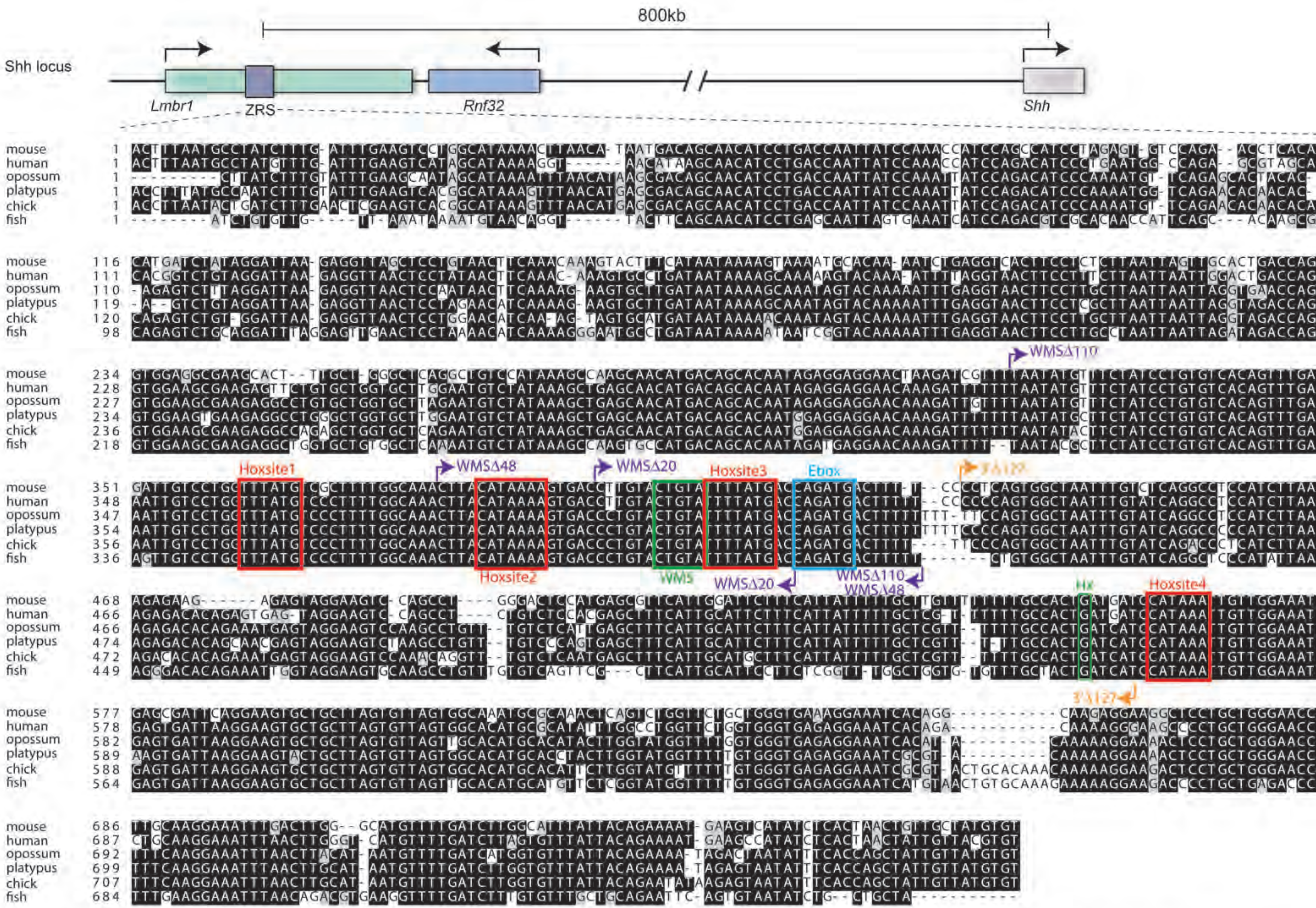

Figure S1. Top line shows a graphical representation of the Lmbr1 to Shh genomic region with the position of ZRS indicated by the grey box. The direction of transcription of the genes is indicated by the arrows. Underneath is the sequence line-up comparing the ZRS in diverse vertebrate species (listed to the left of the sequence) including three different vertebrate classes (mammal, birds and fish) representing >400Myrs of evolution. The sequences for the WMS site and Hx mutation (green) and the Ebox (blue) are boxed. Also boxed in red are the positions of the 4 Hoxsites. The start and end positions of all the large deletions (WMSΔ110, WMSΔ48, WMSΔ20 and 3'Δ127) are indicated.

Figure S2 - relates to Figure 5

Western blots of HOXD proteins and analysis of HOXD12 and D13 binding.

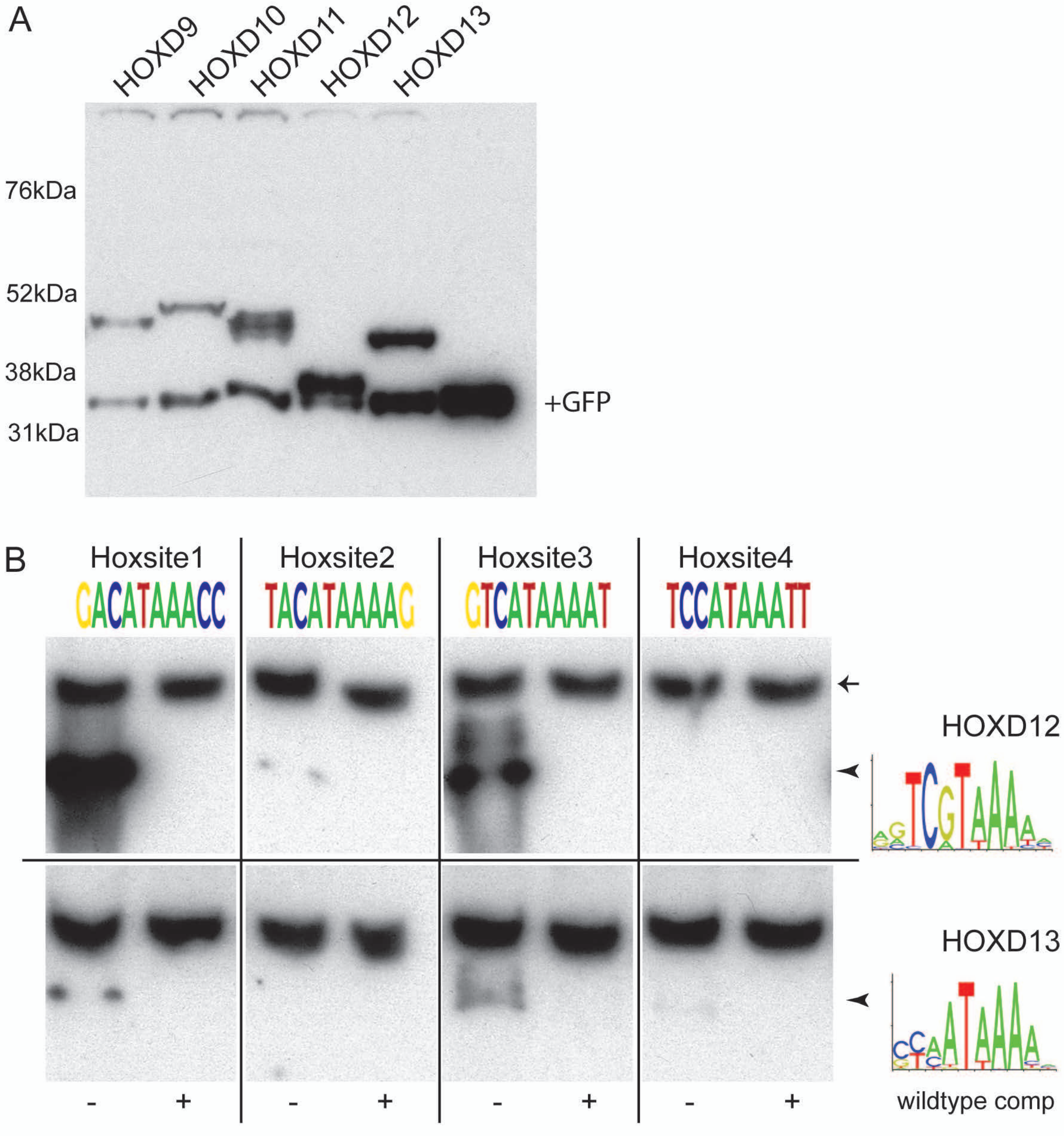

(A) shows a western blot of protein from the IVT reactions probed with an anti HisTag antibody. In addition to the Hox gene containing vector, each reaction included the GFP control vector and a GFP band can be seen in all lanes.

(B) shows the sequence of the Hoxsites1 -4 in the same orientation. The consensus binding sites for the proteins HoxD12 and D13 are shown as position weight matrices under their gene names, adjacent to the relevent EMSAs (D12 in upper panels; D13 in lower panels). For each doublet of EMSAs, the lanes are shown as binding to a labelled Hoxsite oligonucleotide with no competition (lanes marked as -) and with excess of the wildtype oligo as competitor (lanes marked as +). The specific binding is indicated by the arrowheads, while the non-specific band (arrow) indicates the increased length of exposure time necessary, compare with the equivalent band in Figure 4.
